# Metagenome-Assembled Genome Sequence of *Vulcanococcus* sp. Strain Clear-D1, Assembled from a Cyanobacterial Enrichment Culture

**DOI:** 10.1128/MRA.01121-20

**Published:** 2020-12-03

**Authors:** Alexis E. Floback, Kyra M. Florea, J. Cameron Thrash, Eric A. Webb

**Affiliations:** a Department of Biological Sciences, University of Southern California, Los Angeles, California, USA; Georgia Institute of Technology

## Abstract

We report the metagenome-assembled genome sequence of a *Vulcanococcus* sp. binned from a cyanobacterial enrichment culture. The genome contains 39 contigs comprising 2.96 Mbp and is estimated as 100% complete, with a GC content of 63.9% and 3,261 predicted coding genes.

## ANNOUNCEMENT

Formation of harmful algal blooms (HABs) at the volcanically active Clear Lake in California has been reported for decades (1); the HABs are dominated by diazotrophic cyanobacteria, such as *Aphanizomenon* and *Dolichospermum* (2), which are known toxin producers (3, 4). Clear Lake is a source of drinking water for local communities and brings in over 50 million dollars annually through recreational activities and tourism (5). To better understand microbial interactions supporting *Dolichospermum* communities, we performed enrichment culturing and metagenomic sequencing, assembly, and binning. Here, we report the metagenome-assembled genome (MAG) for a species of *Vulcanococcus*, a genus that was only recently isolated from volcanic Lake Albano in Italy (6).

A *Dolichospermum* enrichment was collected at Clear Lake (lat 38.973167, long 122.728089), by a surface bucket tow in August 2019. Hand-picked *Dolichospermum* colonies were cultured for 7 months in 50% BG-11_0_ medium (7)/50% sterile Milli-Q water and incubated at 25°C (100 μmol Q/m^2^/s) on a 12:12-h light/dark cycle, with no NaNO_3_ added to enrich for diazotrophs. Additional medium was added approximately every 2 weeks to maintain growth. Genomic DNA was extracted from the enrichment community with the DNeasy PowerBiofilm kit (Qiagen) following the manufacturer’s instructions but with the addition of five freeze-thaw cycles and a subsequent overnight incubation at 55°C with 25 μl of 20 mg/ml proteinase K and solution C1 from the kit. Isolated DNA was verified with Tris-borate-EDTA (TBE) gel electrophoresis and quantified with NanoDrop UV-visible (UV-Vis) spectroscopy and Qubit spectrofluorometry (Thermo Fisher Scientific, Waltham, MA). Illumina paired-end (PE) 150-bp sequencing (1 Gbp) was performed by Novogene using 300-bp inserts, after library preparation with a NEBNext DNA library preparation kit according to the manufacturer's recommendations. This resulted in 19,844,532 reads. KBase (8) and modules within were used for assembly, as follows. The quality of paired-end reads was checked with FastQC v0.11.5 (9), and sequences were trimmed with Trimmomatic v0.36 (10) with reads under 36 bp being removed. Metagenome assembly was performed with metaSPAdes v3.13.0 (11), and binning was completed with MaxBin v2.2.4 (12). Taxonomy was assigned with GTDB-tk v1.1.0, run with the parameter “classify_wf” and using the release 95 database (13). Default settings were used for all software unless otherwise noted.

One metagenomic bin (Clear-D1) from the enrichment comprised 2,960,550 bp (GC content, 63.9%) in 39 contigs with an *N*_50_ value of 149,920 bp. CheckM v1.0.18 (14) estimated Bin001 as 100% complete with 0.54% contamination, and GTDB-tk classified it as a *Vulcanococcus* sp. The genome was annotated with PGAP v4.11 (15), which predicted 3,083 coding genes, 57 pseudogenes, and 45 noncoding RNA sequences. MetaSanity v3.0 (16) analysis using the FuncSanity module revealed that the *Vulcanococcus* sp. strain Clear-D1 genome has all of the genes required for photoautotrophy but also has genes encoding proteins for sulfide oxidation, sulfur assimilation, and arsenic reduction. Nitrogen fixation genes were missing, unlike in another isolate of this genus (6), and the genome contained predicted genes for thiamine, riboflavin, cobalamin, and retinal biosynthesis. The genome also contained genes for putative ferrous iron transporters and genes for proteins with ferric iron ABC-type substrate-binding capabilities. Thus, this species appears to have competitive nutrient acquisition strategies and interesting capabilities for secondary metabolism that reflect the volcanic activity at Clear Lake.

### Data availability.

This whole-genome shotgun project has been deposited at DDBJ/ENA/GenBank under the accession number JACVZV000000000. The version described in this paper is version JACVZV010000000. The BioProject number is PRJNA657201, and the reads are available at the SRA under accession number SRX8961729.
